# Human RAD51 Protein Forms Amyloid-like Aggregates *In Vitro*

**DOI:** 10.3390/ijms231911657

**Published:** 2022-10-01

**Authors:** Daniel V. Kachkin, Kirill V. Volkov, Julia V. Sopova, Alexander G. Bobylev, Sergei A. Fedotov, Sergei G. Inge-Vechtomov, Oxana V. Galzitskaya, Yury O. Chernoff, Aleksandr A. Rubel, Anna Y. Aksenova

**Affiliations:** 1 Laboratory of Amyloid Biology, St. Petersburg State University, 199034 St. Petersburg, Russia; 2 Research Resource Center “Molecular and Cell Technologies”, Research Park, St. Petersburg State University (SPbSU), 199034 St. Petersburg, Russia; 3 Center of Transgenesis and Genome Editing, St. Petersburg State University, 199034 St. Petersburg, Russia; 4 Institute of Theoretical and Experimental Biophysics, Russian Academy of Sciences, 3 Institutskaya St., 142290 Moscow, Russia; 5 Department of Genetics and Biotechnology, St. Petersburg State University, 199034 St. Petersburg, Russia; 6 Institute of Protein Research, Russian Academy of Sciences, 142290 Pushchino, Russia; 7 School of Biological Sciences, Georgia Institute of Technology, Atlanta, GA 30332-2000, USA

**Keywords:** RAD51, protein aggregation, protein fibrils, amyloid, X-ray diffraction, functional amyloids, amyloidogenesis

## Abstract

RAD51 is a central protein of homologous recombination and DNA repair processes that maintains genome stability and ensures the accurate repair of double-stranded breaks (DSBs). In this work, we assessed amyloid properties of RAD51 *in vitro* and in the bacterial curli-dependent amyloid generator (C-DAG) system. Resistance to ionic detergents, staining with amyloid-specific dyes, polarized microscopy, transmission electron microscopy (TEM), X-ray diffraction and other methods were used to evaluate the properties and structure of RAD51 aggregates. The purified human RAD51 protein formed detergent-resistant aggregates *in vitro* that had an unbranched cross-β fibrillar structure, which is typical for amyloids, and were stained with amyloid-specific dyes. Congo-red-stained RAD51 aggregates demonstrated birefringence under polarized light. RAD51 fibrils produced sharp circular X-ray reflections at 4.7 Å and 10 Å, demonstrating that they had a cross-β structure. Cytoplasmic aggregates of RAD51 were observed in cell cultures overexpressing *RAD51*. We demonstrated that a key protein that maintains genome stability, RAD51, has amyloid properties *in vitro* and in the C-DAG system and discussed the possible biological relevance of this observation.

## 1. Introduction

Amyloids are highly ordered fibrillar protein polymers that are capable of self-assembly and possess an intermolecular cross-β structure. Amyloid fibrils are typically composed of two or more protofilaments or, in some cases, of a single protofilament. Protofilaments are connected to each other in a parallel fashion via their side chains [1]. Each protofilament has a cross-β structure, where β-strands are stacked perpendicular to the fibril axis [2]. Amyloid fibrils can be formed both *in vivo* and *in vitro*.

There are several criteria for distinguishing amyloid fibrils from high-ordered protein fibrils of a non-amyloid nature and also from amorphous protein aggregates [1]. Amyloids usually bind specific dyes, such as Thioflavin T (ThT) and Congo red [3,4]. They are resistant to ionic detergents such as sodium dodecyl sulphate (SDS) and sarcosyl (SLS) [5,6]. Amyloid fibrils demonstrate birefringence under polarized light when stained with Congo red [7,8]. X-ray diffraction of amyloids usually reveals a sharp diffraction signal at 4.7–4.8 Å, corresponding to the distance between the hydrogen-bonded β-strands, and a more diffuse reflection at 10–12 Å, arising from the distance between the β-sheets and depending on the size of side chains [9].

The formation of amyloid aggregates is associated with a diverse group of diseases, the so-called amyloidoses, when insoluble protein fibrils are found in different organs and tissues, and they may underlie the development of a pathology. Some amyloids are associated with neurodegenerative diseases and are found deposited in the nervous system [10]. The elucidation of the role of amyloids and prions in the development and progression of cancer is one of the significant findings of recent years. For instance, it has been shown that the accumulation of the PrP protein in cancer cells promotes the progression of cancer and formation of tumor resistance to various drugs [11]. The tumor suppressor protein, p53, which is frequently inactivated in cancers, is also amyloidogenic [12,13,14]. The list of new amyloids is steadily growing with the unveiling of the amyloid properties of a rising number of proteins and their links with to functional cellular processes or diseases. Some amyloid proteins can interact with nucleic acids and, like p53, are involved in the regulation of DNA repair, DNA replication, or transcription. One example is bacterial RepA, which assembles into amyloid fibrils upon binding to DNA [15]. Another protein, TAR DNA-binding protein 43, also interacts with nucleic acids and is involved in DNA repair [16]. Members of the DNA-damage-inducible 45 (GADD45) protein family have recently been proposed to form amyloid-like aggregates [17].

RAD51 is a central protein of homologous recombination (HR) and homologous DNA repair processes. It is a conserved recombinase that forms filaments on DNA and facilitates the homology search between DNA strands and DNA strand invasion and pairing [18]. RAD51 can form filaments on single-stranded and double-stranded DNA and keeps the DNA in a stretched B-form, thus enabling pairing and the efficient homology search. Double-stranded breaks (DSBs) are the most cytotoxic DNA lesions that can arrest cell division and result in the loss of genetic information. HR and non-homologous end-joining are the two basic pathways of DSB repair that maintain genome integrity. The recombinase activity of RAD51 is implicated in several HR-based DSBs repair routes, such as gene conversion, synthesis-dependent strand annealing and RAD51-dependent break-induced replication [19,20]. Certain RAD51 activity is vital for HR and genome stability. The modulation of HR activity can affect ability of the cell to cope with different DNA lesions and ensure their accurate repair.

The filaments of RAD51 on DNA have a helical symmetry and are similar to RecA filaments [21,22]. *In vivo*, RAD51 can accumulate to create nuclear foci that have a dot appearance, and their generation correlates with DNA damage and the cell cycle [23,24]. RAD51 has a natural tendency to form heterogeneous aggregates, but its filament behavior in the cell seems to be tightly controlled [19,25,26]. This study demonstrates, for the first time, that RAD51 can form amyloid-like aggregates *in vitro* and in the bacterial C-DAG system. RAD51 aggregates exhibit all the properties of amyloids, including resistance to detergents, birefringence in polarized light, binding to ThT and the X-ray diffraction pattern typical for amyloids.

## 2. Results

### 2.1. RAD51 Protein Forms Aggregates In Vitro That Are Resistant to SDS

**Figure 1:**
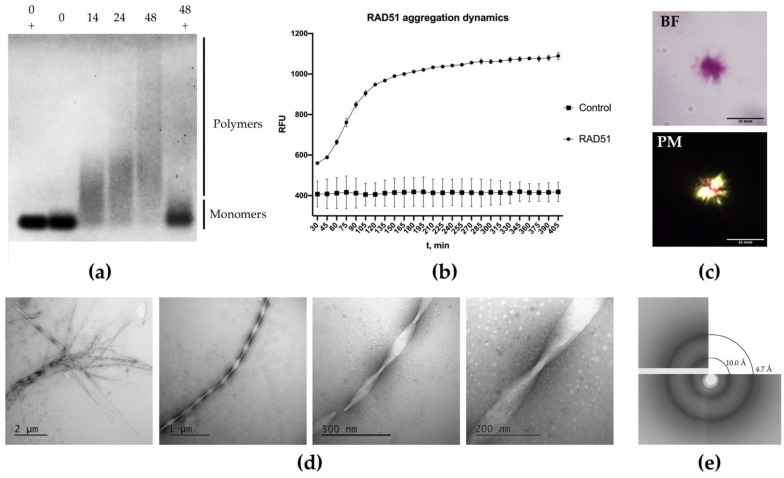
Analysis of RAD51 aggregation *in vitro*. (**a**) RAD51 forms SDS-resistant aggregates *in vitro* and its aggregation increases over time. SDD-AGE of RAD51 monomers and aggregates: Agarose 1%/0.1% SDS gel is presented. Numbers above the gel (0, 14, 24, 48) correspond to the incubation time (hours), “+” reflects samples that were boiled before loading on the gel, and all samples were treated with 1% SDS prior to loading on the gel. The gel was stained as described in MM, and monomers and polymers are indicated. (**b**) Aggregation dynamics of RAD51 monomers in the presence of ThT. RFU—relative fluorescence unit; t, min—incubation time (minutes). (**c**) RAD51 aggregates bind to Congo red and show purple staining and are birefringent in polarized light. BF—bright field, PM—polarized light. (**d**) Structure of RAD51 fibrils analyzed by TEM. The samples were prepared as described in MM. (**e**) X-ray diffraction of RAD51 aggregates formed *in vitro*, which exhibit reflections at ~4.7 Å and ~10 Å that can be ascribed to their amyloid cross-β sheet structure.

RAD51 is known to be prone to aggregation, although no one has characterized the aggregates or reported SDS-resistant RAD51 aggregates to the best of our knowledge. We purified the human RAD51 protein from *E. coli* as a fusion with a SUMO-tag that was cleaved away at the end of the procedure, leaving the intact RAD51 protein (Figure S1a). We found that RAD51 easily formed aggregates *in vitro* upon agitation. Then, we decided to conduct an assay to assess whether the formed RAD51 aggregates were detergent-resistant. The ability to form detergent-resistant aggregates is one of the main characteristics of amyloid fibrils that allows researchers to effectively distinguish between amyloid and non-amyloid protein complexes [27]. RAD51 samples were incubated at 37 °C in conditions that included samples’ shaking, as described in the Materials and Methods, aliquots were collected at different time points and analyzed by semi-denaturing detergent agarose gel electrophoresis (SDD-AGE). Specifically, we treated the samples with 1% SDS, and then they were run on 1% Agarose/0.1% SDS gel. As a control, RAD51 monomers and RAD51 aggregates after 48 h of incubation were boiled for 5 min and run on the same gel. As seen in Figure 1a and Figure S1b, RAD51 aggregates do not break down into monomers or oligomers after exposure to 1% SDS and are visible on the gel as extended smears. These smears appear after 14 h of incubation, and their amount and size increase with time (Figure 1a). Monomeric RAD51 (no incubation with shaking) does not form such smears and runs at the bottom of the gel, appearing as sharp-cut bands. Thus, the RAD51 aggregates we obtained were SDS-resistant, which implied that they may represent amyloid-like aggregates.

### 2.2. RAD51 Binds to Thioflavin T during Aggregation

Amyloid fibrils can be readily detected using ThT, a molecule that emits fluorescence when bound to amyloids [28]. The use of ThT allows us to detect the presence of amyloid aggregates in a solution and to quantify the rate of aggregation and the growth of fibrils. The study of the RAD51 aggregation dynamics showed that the monomeric RAD51 started to aggregate after the start of the experiment (Figure 1b), and fibril growth continued for the next 6 h with a standard sigmoid curve that is also typical for amyloidogenic proteins [28,29,30]. In the control sample without monomers of RAD51, there was no increase in the level of fluorescence. Thus, during RAD51 aggregation, we detected increasing binding to the amyloid-specific dye Thioflavin T. We also showed that RAD51 aggregation can be seeded by preformed aggregates (Figure S2). This indicates that RAD51 aggregation is amyloid-like.

### 2.3. RAD51 Aggregates Bind to Congo Red and Glow under Polarized Light

The ability to bind to the amyloid-specific dye Congo red and glow in polarized light is characteristic of amyloid fibrils [8]. The RAD51 aggregates obtained *in vitro* were stained with Congo red and the samples were examined in brightfield and polarized lights. As seen in Figure 1c, RAD51 aggregates demonstrate purple staining in the brightfield and are birefringent under polarized light. Howie A. thoroughly described that Congo-red-stained amyloids typically demonstrate mixed color birefringence [7]. We detected a mixture of yellow, green, blue and red colors in our samples. A similar picture was observed when RAD51, was produced as an extracellular protein in the bacterial curli-dependent amyloid generator (C-DAG) system (Figure S3). This bacteria-based system provides a simple and efficient means of distinguishing proteins with inherent amyloid-forming properties from those that do not readily undergo conversion to an amyloid state [31].

Thus, again, we can conclude that RAD51 aggregates demonstrate typical amyloid-like traits.

### 2.4. RAD51 Aggregates Resistant to 1% SDS Have a Fibrillar Structure Visible under an Electron Microscope

Our experiments demonstrated that RAD51 aggregates, *in vitro*, possess some key features of the amyloids, such as resistance to ionic detergents, binding to ThT and birefringence when stained with Congo red. Thus, we next aimed to study the structure of RAD51 aggregates by transmission electron microscopy (TEM). RAD51 aggregates were treated with 1% SDS for 10 min prior to applying to the electron microscopy grid, then washed and counterstained with 1% uranyl acetate. Upon the TEM analysis, we found that the aggregates in the samples were represented by large extended twisted fibrils which were about 25–50 nm thick and at least 1 μM long and were combined together in thick (~1 μM) fibril bundles (Figure 1d). The structure we observed resembles the typical amyloid structures, where the fibrils are needle-like and unbranched, consisting of several protofilaments of a few nanometers in width and around a micrometer in length, which are laterally bundled [2]. The secretion of the CsgA_ss_-RAD51 protein in the C-DAG system also led to the formation of fibrils, visualized by TEM (Figure S3).

### 2.5. X-ray Diffraction of RAD51 Fibrils Shows a Typical Cross-β Diffraction Pattern Typical for Amyloids

To further investigate the structure of the RAD51 fibrils, we performed X-ray diffraction on an air-dried sample of RAD51 fibrils. This analysis revealed sharp circular X-ray reflections at 4.7 Å and 10 Å (Figure 1e). These results are typical for amyloid fibrils with a cross-β sheet quaternary structure. The diffraction pattern at ~4.7–4.8 Å is dominated by an intense reflection derived from the mean separation of the hydrogen-bonded β-strands that are arranged perpendicular to the fiber axis in the cross-β structure, and the diffraction pattern at 10–11 Å reflects the distance between the β-sheets [2,32]. The fibrillar morphology, cross-β structure and characteristic tinctorial properties are universally accepted as the hallmarks of an amyloid structure, and any given protein aggregate displaying all three of them can be classified as an amyloid [33,34,35,36,37,38]. Considering the data we obtained, we declare that the human RAD51 protein can form amyloid structures *in vitro*.

### 2.6. Aggregation of RAD51 in HEK293T Cells

**Figure 2:**
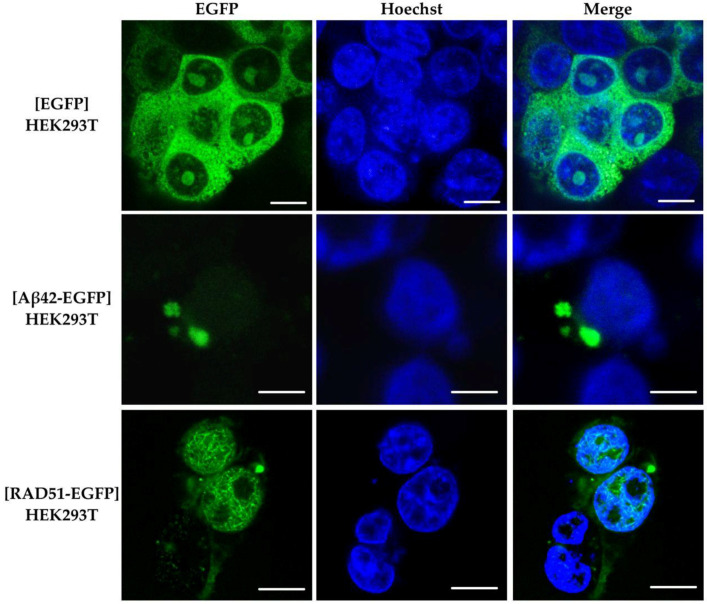
RAD51 aggregation in human cells. Confocal microscopy of HEK293T cells with the overexpression of the *RAD51-EGFP*. EGFP—GFP channel, Hoechst—nuclear staining by Hoechst 33342. [EGFP]/[Aβ42-EGFP]/[RAD51-EGFP]—fusion proteins that were overproduced in the HEK293T cell line. Scale bar—20 µm.

RAD51 is known to form foci in the nucleus that have a dot-like appearance and represent RAD51 accumulation at the sites of DNA damage [23]. RAD51 foci and higher-order nuclear structures of up to 20–30 µm in length have been observed upon *RAD51* overexpression [39], although this finding has not been confirmed by other studies [40]. To investigate the possibility of RAD51 aggregation in mammalian cells, HEK293T cells were transfected with pLenti-hRAD51(FL)-EGFP plasmid, expressing the *RAD51-EGFP* fusion. The plasmids pLenti-CMV-EGFP (producing monomeric EGFP protein, which is not prone to aggregation) and pLenti-CMV-hAβ42-EGFP (producing human Aβ42 peptide, which is capable of amyloid aggregation) were used as controls. The overexpression was driven by the constitutive *CMV* promoter. As can be seen in Figure 2, when the RAD51-EGFP is overproduced in HEK293T cells, visible round-shaped aggregates appear in the cytoplasm. The negative control, the cells with EGFP overproduction, demonstrated a diffuse distribution of the fluorescent protein in the cytoplasm. HEK293T cells with an overproduction of Aβ42-EGFP fusion protein, which formed distinct aggregates in the cytoplasm, were used as a positive control. This indicates the ability of the human RAD51 protein to aggregate in the cytoplasm of mammalian cells upon overproduction. We also observed high-order structures formed by RAD51-EGFP in the nucleus, which may resemble the structures described earlier in the case of RAD51 overproduction [39]. Note that these structures were not observed when anti-RAD51 antibodies were used for the detection (Figure S4), which can possibly be explained by the poor penetrance of the nuclei in the case of the antibodies and, consequently, a weaker signal compared to EGFP detection. An alternative explanation is that these structures are not recognized by anti-RAD51 antibodies. We also observed RAD51 enrichment around the nucleus when anti-RAD51 antibodies were used (Figure S4).

### 2.7. Bioinformatic Analysis of RAD51’s Amyloidogenic Potential

**Figure 3:**
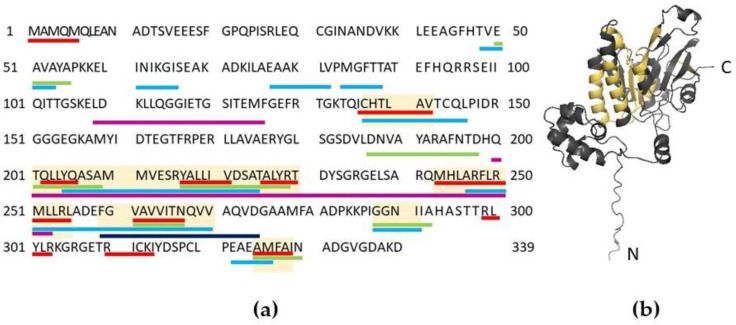
Representation of the amyloidogenic regions in RAD51. (**a**) Analysis performed by FoldAmyloid (red), Waltz (green), AGGRESCAN (light blue), ArchCandy (magenta) and PASTA 2.0 (dark blue). Light yellow boxes represent amyloidogenic regions predicted by at least two algorithms that are ≥5 a.a. long. (**b**) The 3D model of the RAD51 protein was generated using AlphaFold2 [50] and visualized with PyMol (pymol.org). The amyloidogenic regions predicted by at least two algorithms (≥5 a.a. long) are shown in yellow.

We used five different programs to analyze the amyloidogenic properties of RAD51: Pasta 2.0 [41], FoldAmyloid [42], AGGRESCAN [43], Waltz [44] and ArchCandy [45]. The amyloidogenic regions in RAD51 are more concentrated in the second half of the protein sequence (Figure 3a), and most of them are located in the region from 200 a.a. to 300 a.a. The conserved RecA-homology domain of RAD51 (spanning 96–314 a.a.) therefore carries most of the detected amyloidogenic regions. The 3D modelling of the full-length RAD51 monomer demonstrated that amyloidogenic regions cover the central β-regions, which are involved in the formation of a large β-sheet, and also cover several α-helices (Figure 3b). Notably, the amyloidogenic regions overlap with several functional RAD51 domains. RAD51 has Walker A and B motifs (127–134 a.a. and 218–222 a.a., respectively), which are important for ATP binding and hydrolysis [19]. The Walker B motif overlaps with the amyloidogenic regions predicted by the four algorithms. The nuclear export signal at 245–260 a.a. [46] falls in another amyloidogenic region. Residues L205, R247, R250 and L255 form a LFDE-binding pocket that is essential for the interaction with the BRC-repeats of BRCA2 [47]. Other interesting regions are located at 184–257 a.a., representing a domain responsible for the interaction with the partner of BRCA2, the tumor-suppressor PALB2 [48], and at 125–220 a.a., involved in the interaction with p53 [49]. Thus, we hypothesize that the interaction of RAD51 with its partners, such as BRCA2, or RAD51 nuclear localization may be affected by the amyloid aggregation of RAD51 or *vice versa*.

## 3. Discussion

Amyloid fibrils are classified as supramolecular polymers that have a fibrillar appearance and cross-β structure, with the β-strands stacked perpendicularly to the long axis of the fibril [2]. These structures can naturally occur in living organisms as part of a physiological process or pathology. The number of amyloid proteins is steadily growing, implicating this amazing phenomenon in various cellular processes and pathological conditions. It is known that these proteins different in their amino acid compositions and possessing different structures are capable of forming amyloid fibrils that demonstrate a unique set of characteristics. These include an unbranched fibrillar morphology, a cross-β structure and birefringence upon binding to Congo red and to ThT [33].

In this work, we demonstrated, *in vitro*, that a key protein that maintains genome stability, RAD51, has the properties of an amyloid protein. We showed that RAD51 forms detergent-resistant aggregates that have an unbranched fibrillar structure and are stained with amyloid-specific dyes, such as ThT and Congo red. When stained with Congo red, RAD51 aggregates have an increased birefringence under polarized light. Our X-ray diffraction analysis demonstrated that RAD51 fibrils possess a cross-β structure that is characteristic of amyloid proteins. Additionally, we found that RAD51 forms fibrillar structures in the bacterial C-DAG system that can distinguish amyloidogenic proteins from proteins with no amyloid properties [31].

RAD51 is an oligomeric protein with several parallel and antiparallel β-strands in its structure. The β-strands’ pairing seems to play an essential role in the ability of RAD51 to form helical nucleoprotein filaments on DNA substrates and can provide surface interactions with its partners, such as BRCA2 [25]. The ability of RAD51 to form amyloid fibrils *in vitro* is a new feature, which has not previously been described. We have also shown that RAD51, upon overproduction, forms aggregates in the cytoplasm. Though the nature of these aggregates is not entirely clear, we can assume that their occurrence might be related to the amyloidogenic properties of RAD51. Many amyloid proteins, when overproduced in cells, form aggregates in the cytoplasm [51]. In some cases, such aggregation may lead to protein sequestration and functional loss [52].

The homologous recombination DNA repair system plays a fundamental role in the stability of the genome. This system enables the error-free repair of DSBs, which pose the most severe danger to the cell. It also allows for the repair of intermolecular cross-links and DNA adducts, which can stall the progression of DNA polymerases. The homologous recombination repair system is extremely important when cells are actively dividing or are exposed to DNA-damaging agents. The sequestration of RAD51 and, possibly, its interacting partners in cytoplasmic aggregates may lead to its functional deficiency and affect homologous recombination DNA repair and genome stability.

Genome instability, in many tumors, is linked to mutations in the genes encoding components of the DNA recombination repair system. Some cancers, including ovarian and breast cancers, are known to be HR-deficient and often have mutations in the RAD51 paralogs or RAD51 mediators. These cancers use alternative repair pathways (e.g., poly(ADP-ribose) polymerase, PARP) to maintain the stability of genetic material. RAD51 is implicated in the resistance of cancer cells to PARP inhibitors, therefore representing a perspective target for anticancer therapies [53,54,55].

Remarkably, the mechanisms of protein inheritance may enable fast adaptation to the changing environment and the spreading of the corresponding phenotype to the adjacent cells and tissues. In fact, amyloids and amyloid proteins have already been associated with various tumors [56]. Some of these proteins, such as p53, control genome stability and the cell cycle progression. Even more striking is the evidence suggesting that the aggregation of p53 induces a prion-like determinant in a yeast model, indicating that p53 aggregates might be infectious and spread across different cells [57]. The amyloid proteins SAA are positively correlated with the cancer stage in lung cancer, breast cancer and melanoma [58,59,60,61]. It was suggested that SAA may enhance cancer cell proliferation and migration through the regulation of inflammation [62,63]. The amyloid protein S100A9, which is one of key factors promoting neuroinflammation [64], plays a role in prostate cancer invasion and is associated with disease progression [65]. This amyloidogenic protein was also found to be up-regulated in other cancers, including breast cancer, colon cancer, hepatocellular carcinoma, gastric cancers and others [66]. Tau amyloid is associated with ganglioglioma [67], IAPP with neuroendocrine tumors [68], the N-terminus of prolactin with pituitary adenoma [69] and ODAM with odontogenic tumors [70,71].

Further studies are required in order to elucidate the biological significance of RAD51 aggregation and its role in genome instability and tumorigenesis.

## 4. Materials and Methods

### 4.1. Bacterial Strains and Cultivating Conditions

*Escherichia coli* strain XL10-Gold (Stratagene, La Jolla, CA, USA) was used for plasmid construction and plasmid DNA amplification. Rosetta *E. coli* strain (Sigma-Aldrich, St. Louis, MO, USA) was used for the RAD51 production. VS39 *E. coli* strain was used for the C-DAG experiments [31]. Luria-Bertani (LB) broth and LB agar plates were used for the *E. coli* cultivation. Ampicillin was added to LB agar medium at a final concentration of 100 µg/mL for the selection of ampicillin-resistant bacterial transformants (Green and Sambrook, 2012). Bacteria were cultivated at 37 °C in a shaker-incubator ES-20/60 (Biosan, Riga, Latvia) at 180 rpm.

### 4.2. Cell Cultures

Human embryonic kidney 293T (HEK293T) cells were used to analyze the possibility of the aggregation of the human RAD51 protein in the mammalian cells. HEK293T were maintained in DMEM medium (Capricorn Scientific, Ebsdorfergrund, Germany) supplemented with 10% fetal bovine serum (FBS) (Life Technologies (Gibco), Carlsbad, CA, USA) and 1× Gentamycin (Life Technologies (Gibco), Carlsbad, CA, USA).

The IMR-32 cell line was used to obtain the human *RAD51* cDNA sequence. IMR-32 cells were maintained in RPMI-1640 (Life Technologies (Gibco), Carlsbad, CA, USA) medium supplemented with 10% fetal bovine serum (FBS) (Life Technologies (Gibco), Carlsbad, CA, USA) and 1× Gentamycin (Life Technologies (Gibco), Carlsbad, CA, USA). Both cell lines were cultivated inside a humidified incubator, Galaxy 14 S (Eppendorf/New Brunswick Scientific, Edison, NJ, USA), at 37 °C and in 5% CO_2_.

### 4.3. Plasmids

The *RAD51* full-length cDNA PCR product was obtained from human cDNA (neuroblastoma cell line, IMR-32) with Takara ExTaq (Takara Bio USA Inc., San Jose, CA, USA) using the primers hRAD51NMFOR (TAACAATTGATGGCAATGCAGATGCAGCTTGAA) and hRAD51STOP (TTAGCGGCCGCTTAGTCTTTGGCATCTCCCACTCCATCT). The PCR products were cut with *Mfe*I and *Not*I and inserted into the pCUP-Sup35NM-Ab(1-42) plasmid [72], where the Aβ sequence was excised with *Eco*RI and *Not*I. The *RAD51* was sequenced, and the encoded amino acid sequence corresponded to the canonical isoform Q06609-1 (Uniprot, accessed on 11 May 2022). This full-length *RAD51* sequence was amplified with Takara ExTaq (Takara Bio USA Inc, San Jose, CA, USA) using the primers hRAD51FbsmBI (TACGTCTCTAGGTATGGCAATGCAGATGCAGCTTGAA) and hRAD51RXbaI (ATTCTAGATTAGTCTTTGGCATCTCCCACTCCAT). The PCR product was cut with *BsmB*I and *Xba*I and cloned into pE-SUMO-pro plasmid (LifeSensors, Malvern, PA, USA), cut with *Bsa*I. This generates the pE-SUMO-hRAD51FL plasmid carrying the *RAD51* sequence, fused in frame with the yeast *SMT3* sequence (SUMO), and the sequence encoding 6×His-Tag (both are on the N-terminus of the protein). This construct allows for the efficient expression of the RAD51 protein in a soluble form in *E. coli* and its purification.

To study the aggregation of the human RAD51 protein in mammalian cells, plasmid pLenti-CMV-hRAD51(FL)-EGFP was obtained for the transfection of the HEK293T cells. This plasmid carries the chimeric *RAD51* gene, which was fused with *EGFP* and placed under the control of the *CMV* promoter. To obtain the plasmid, the pLenti-CMV-GFP Hygro (656-4) vector [73] carrying *EGFP* under the control of the *CMV* promoter was digested with the restriction endonucleases *BamH*I and *Xba*I and ligated with the *RAD51* PCR product obtained with the primers hRAD51-XbaI-For (CATCTAGAATGGCAATGCAGATGCAGC) and hRAD51(FL)-BamHI+2-Rev (CAGGATCCGCGTCTTTGGCATCTCCCAC) and digested with *Xba*I and *BamH*I. The pLenti-CMV-Aβ42-EGFP plasmid was obtained for the transfection of the HEK293T cells and the demonstration of amyloid aggregation in the human cells. To obtain the plasmid, a pLenti-CMV-GFP Hygro (656-4) vector [73] carrying *EGFP* under the control of the *CMV* promoter was digested with the restriction endonucleases *BamH*I and *Xba*I and ligated with the Aβ42 PCR product obtained with primers the Abeta-XbaI-For (CATCTAGAATGGATGCAGAGTTCCGACATG) and Abeta-BamHI+2-Rev (TAGGATCCAACGCTATGACAACACCG) and then similarly digested with *Xba*I and *BamH*I. The pCup-Sup35N-Ab42 plasmid [72] was used as a matrix for the Aβ42 amplification.

To study RAD51 aggregation in the C-DAG system, we used the pVS72 plasmid encoding the CsgAss-Sup35NM chimeric protein [31]. This plasmid was used for the construction of the pVS-C-DAG-hRAd51(FL) plasmid, encoding RAD51 protein fused with the bipartite signal sequence CsgA_ss_. *RAD51* was amplified with the hRAD51-NotI-For (AAGCGGCCGCAGCAATGCAGATGCAGCTTGAAG) and hRAD51(FL)-XbaI-Rev (TATTCTAGATTAGTCTTTGGCATCTCCCAC) primers and, next, this fragment was digested with *Not*I and *Xba*I and was inserted into the pVS72 vector, similarly cut with *Not*I and *Xba*I.

The accuracy of the obtained plasmids was confirmed by restriction analysis and by sequencing according to the Sanger method.

### 4.4. Mammalian Cell Transfection

The transfection of the HEK293T human cells was performed using TurboFect Transfection Reagent (Thermo Fisher Scientific, Waltham, MA, USA). The day before transfection, the HEK293T cells were sub-cultured in a 24-well plate at a seeding density of 1 × 10^5^ cells per well (counting was carried out using a hemocytometer) and incubated overnight in DMEM medium (Capricorn Scientific, Ebsdorfergrund, Germany) supplemented with 10% FBS (Life Technologies (Gibco), Carlsbad, CA, USA), 1% GlutaMAX (Life Technologies (Gibco), Carlsbad, CA, USA) and 10 µg/mL gentamicin (Life Technologies (Gibco), Carlsbad, CA, USA). After 18 h of incubation, 100 μL of the transfection mixture was prepared. A total of 1 μg of plasmid DNA was added to the Opti-MEM medium (Life Technologies (Gibco), Carlsbad, CA, USA) and thoroughly mixed by shaking on a Vortex Genius 3 shaker (IKA-Werker, Staufen im Breisgau, Germany). A total of 2 µL of TurboFect Transfection Reagent was added to the mixture and again thoroughly mixed on a shaker. The resulting mixture was incubated for 15 min at room temperature. The medium was removed from the cells and 900 µL of fresh standard DMEM medium was added. The transfection mixture was added to the well and mixed by the gentle rocking of the plate. The cells were incubated for 18 h at 37 °C in a CO_2_ incubator with 5% CO_2_ in humidified conditions. After 18 h, the medium with the transfection mixture was removed and fresh standard medium was added [74].

### 4.5. Immunofluorescence

The HEK293T cells were seeded on round coverslips of approximately 7 × 10^4^/cm^2^, which were pre-treated with type I collagen (Institute of Cytology of the Russian Academy of Sciences, St. Petersburg, Russia). The next day, the cells were fixed with 2.5% formalin (Sigma-Aldrich, St. Louis, MO, USA) for 10 min at room temperature. The fixed cells were washed to expel the formalin with phosphate buffered saline (PBS), pH 7.4 (Sigma-Aldrich, St. Louis, MO, USA), after which they were treated with 0.1% Triton X-100 (Amresco, Solon, OH, USA) solution for 15 min at room temperature. Next, the cells were washed with PBS-T (PBS with 0.05% Tween-20), pH 7.4, and hybridized with polyclonal antibodies to RAD51 (ab88572) (Abcam, Cambridge, UK) at a dilution of 1:50 for 12 h at +4 °C. Then, the cells were washed 3 times with PBS-T and hybridized with secondary goat anti-mouse antibodies conjugated with Alexa Fluor 647 (ab150115) (Abcam, Cambridge, UK) at a dilution of 1:500 for 1 h at room temperature. After hybridization with the secondary antibodies, the cells were washed 3 times with PBS-T and the cell nuclei were stained with Hoechst 33342 Ready Flow (Thermo Fisher Scientific, Waltham, MA, USA). Hoechst 33342 was diluted at a ratio of 1:100 in PBS-T, and the cells were incubated at room temperature for 5–10 min before being analyzed by fluorescence microscopy.

Cells were analyzed using the confocal microscope Leica TCS SP5 (Leica Microsystems GmBH, Wetzlar, Germany) at the Resource Centre “Chromas”, SPBU. An argon laser with a wavelength of 488 nm was used to detect EGFP-containing chimeric protein constructs, and the signal was detected in the range of 500–530 nm. Alexa 647 was detected using a helium-neon laser with a wavelength of 633 nm, and the signal was detected in the range of 660–680 nm. A UV laser with a wavelength of 405 nm was used to detect the nucleus dyed with Hoechst.

### 4.6. In Vitro Protein Expression and Purification

Rosetta *E. coli* cells were transformed with pE-SUMO-hRAD51FL plasmid and the cells were grown in conical flasks (500 mL) in LB medium with ampicillin until OD*_600_*= 0.8. The *RAD51* expression was induced with 1 mM IPTG at 20 °C overnight. The next day, the cells were harvested and resuspended in 10× excess (*v*/*w*) of lysis buffer (25 mM Tris-HCl pH 8.0, 500 mM NaCl, 5% glycerol), and then lysozyme (1 mg/mL final concentration) and NP-40 (0.2% final concentration) were consequently added. The cells were stirred on ice for 15 additional minutes, frozen in liquid nitrogen and stored in −80 °C for at least 1 h. After thawing PMSF was immediately added (1 mM final concentration) to the cells and the lysate was sonicated on a Bandelin SONOPULS HD 2070 homogenizer (BANDELIN electronic GmbH & Co., Berlin, Germany) at 2 kHz 6 times for 30 s, with a 1 min interval on ice between each sonication. After that, the lysate was centrifugated at 15,000× *g* for 30 min. The supernatant was transferred into a fresh tube and Imidazole was added to a final concentration of 10 mM.

The supernatant was filtered through 45 μM Millipore filters and loaded on a 5 mL HisTrap HP column (GE Healthcare, Boston, MA, USA) equilibrated with buffer A: 25 mM Tris-HCl pH 8.0, 500 mM NaCl, 0.1% NP-40, 10 mM Imidazole. Then, the column was washed with the same buffer and the protein was eluted with 20CV linear gradient until 500 mM of Imidazole concentration was obtained. The peak fractions of the RAD51 protein were collected and combined. The buffer in the sample was exchanged for Buffer B: 25 mM Tris pH 8.0, 150 mM NaCl, 0.1% NP-40, 10% glycerol on HiTrap HP with Sephadex G25 (GE Healthcare, Boston, MA, USA) 4 × 5 mL desalting columns. Then, the DTT was added with a final concentration of 1 mM. The Ulp1 protease fragment was added to the sample in the proportions of 10:1 (10 parts of RAD51: 1 part of Ulp1) to cut away the 6×His-SUMO tag. The sample was incubated overnight at 4 °C and centrifuged, Imidazole was added to the sample at up to 10 mM and the sample was loaded on a 5 mL HisTrap HP column (GE Healthcare, Boston, MA, USA) equilibrated with buffer A. The flowthrough (FT) and the wash (in the same buffer) were collected. The protein-containing fractions from the FT and wash were pooled together. The buffer was exchanged for buffer B by dialysis.

### 4.7. In Vitro Protein Aggregation

For the aggregation assay, the RAD51 protein was diluted in buffer B. Unless indicated otherwise, the protein concentrations used in experiments were 0.1 mg/mL, estimated using the Qubit Protein Assay (Invitrogen, Waltham, MA, USA). RAD51 aliquots of 300 μL each were incubated in the Thermomixer (Eppendorf, Hamburg, Germany) for 1 min at 300 rpm alternated with 15 min of rest. The temperature of the incubation was 24 °C or 37 °C. We noted more effective aggregation at 37 °C and used this temperature for most of the experiments. The aggregation was monitored by SDD-AGE after 14, 24 and 48 h. For some experiments, we used longer incubation times of 72 and 96 h. For SDD-AGE, the 20–30 μL protein aliquots were sampled, mixed proportionally with 4× Laemmly Buffer with β-ME and 1% SDS (final concentration) and incubated at room temperature for 10 min. Then, the samples were loaded on 1% Agarose gel with 0.1% SDS and run at 80 V for 2 h. PageRuler or Spectra BR ladders (Thermo Fisher Scientific, Waltham, MA, USA) were used to monitor the band sizes. For the monomer controls, we used purified protein aliquots sampled before aggregation and samples after boiling. The gels, after electrophoresis, were washed in H_2_O and stained with Coomassie G250 for 20 min, then washed in ethanol-acetic acid (H_2_O, 40% ethanol, 10% acetic acid) until the colored bands became visible and could be analyzed. Otherwise, proteins were transferred to a membrane, and Western blotting was performed and the detection was carried out with polyclonal antibodies to RAD51 (ab88572) (Abcam, Cambridge, UK) and secondary ECL Peroxidase labelled anti-mouse antibody (NA931VS) (GE Healthcare, Chicago, IL, USA).

### 4.8. RAD51 Aggregation Dynamics

To assess RAD51 aggregation *in vitro*, 7.6 µM of the RAD51 monomers was placed in Tris buffer saline (TBS) with 0.01% NP-40, 10% glycerol and 10 µM ThT. A buffer without the addition of a monomeric protein was used as a control. The samples were incubated for 400 min at 37 °C with periodical agitation (100 rpm) for 10 min, with 5-min pauses, when the fluorescence E_ex_ = 441 nm/E_em_ = 486 nm was measured. All measurements were carried out in triplicate on the Spark™ 10M multimode reader (Tecan Life Sciences, Männedorf, Switzerland). Reactions were prepared on ice to achieve a final volume of 200 µL. In the seeding experiments, we used preformed RAD51 aggregates (final concentration 0.01 mg/mL) as seeds for the RAD51 monomers (the monomer concentration was 1.5 µM). The aggregation was performed at 37 °C, followed by fluorescence reading at Eex = 441 nm/Eem = 486 nm.

### 4.9. Microscopy Assays (TEM, Polarized, Confocal)

Protein samples were initially treated with 1% SDS solution for 10 min at room temperature, after which they were applied to a grid coated with a formvar film (Agar Scientific, Stansted, UK). The samples on the mesh were washed twice with milliQ water, after which they were counterstained with 1% uranyl acetate aqueous solution for 1 min. The data were obtained using a transmission electron microscope (TEM) Jeol JEM-2100HC (Jeol Ltd., Tokyo, Japan).

The Congo red staining and birefringence were analyzed by Leica DMI6000. The stock solution of the Congo red dye (Sigma-Aldrich, St. Louis, MO, USA) was prepared by dissolving it in milliQ water until a final concentration of 250 mg/mL was achieved and then filtered 3 times through 45-μm Millipore filters. RAD51 aggregates were concentrated by centrifugation at 20,800× *g* for 60 min and then applied to a glass slide, air-dried and stained with water Congo red solution.

The RAD51 aggregation in the HEK293T cells was analyzed using a confocal microscope, Leica TCS SP5 (Leica, Wetzlar, Germany).

### 4.10. RAD51 X-ray Diffraction Analysis

The RAD51 fibrils for the X-ray diffraction (XRD) analysis were prepared using ~1.4 mg of purified RAD51 protein. The aggregation was performed using multiple 500 μL samples in 1.5 mL tubes in the Thermomixer (Eppendorf, Hamburg, Germany) at 37 °C, with 300 rpm shaking for 1 min and 15 min rest. After 72 h, all the protein samples were centrifuged at 20,800× *g* for 1.5 h, and then the supernatant was discarded and the pellet was resuspended in 500 μL H_2_O and combined together in one tube, then centrifuged again at 20,800× *g* and washed with H_2_O. This step repeated twice. Then, the pellet was dried, dissolved in 30 μL of water and used for the TEM control and for XRD. Droplets of these preparations were placed between the ends of wax-coated glass capillaries (approximately 1 mm in diameter) separated at a distance of 1.5 mm. Fiber diffraction images of RAD51 were collected on a XtaLab Synergy S (Rigaku, Tokyo, Japan) instrument with a HyPix detector and a PhotonJet microfocus X-ray tube using Cu Kα (1.54184 Å) radiation. The images were prepared using the CrysAlisPro (Agilent Technologies, Inc., Oxfordshire, UK) data reduction package. The experiments were carried out at a 2° phi rotation and the exposure time was 60 s.

### 4.11. RAD51 Aggregation Analysis in the C-DAG System

The analysis of the RAD51 fibril formation in the bacterial C-DAG system was performed as described in [75,76]. *E. coli* VS39 strain was transformed with the pVS-C-DAG-hRAd51(FL) plasmid coding for the RAD51 protein fused to the CsgA_ss_ signal sequence. VS39 transformants with the pVS72 and pVS105 plasmids encoding the CsgA_ss_-Sup35NM and CsgA_ss_-Sup35M proteins were used as positive and negative controls in the amyloid generation experiments, respectively. Bacterial cultures expressing recombinant proteins were analyzed through Congo red staining and electron microscopy.

## 5. Conclusions

We have shown that a key protein that maintains genome stability, RAD51, has amyloid properties *in vitro* and in the bacterial C-DAG system. This observation opens up new possibilities how RAD51 can facilitate its function in DNA repair and recombination. We observed formation of cytoplasmic RAD51 aggregates in mammalian cells in addition to nuclear structures observed by us and others earlier. RAD51 sequestration in aggregates in the cytoplasm may lead to its functional deficiency; alternatively, RAD51 in its amyloid state may acquire new yet not known functions. Further studies are needed to elucidate the biological role of RAD51 amyloid-like aggregation and its possible implication in DNA repair, genome stability and tumorigenesis. 

## Supplementary Materials

The following supporting information can be downloaded at: https://www.mdpi.com/article/10.3390/ijms231911657/s1.

## References

[1] Benson M.D., Buxbaum J.N., Eisenberg D.S., Merlini G., Saraiva M.J.M., Sekijima Y., Sipe J.D., Westermark P. (2020). Amyloid Nomenclature 2020: Update and Recommendations by the International Society of Amyloidosis (ISA) Nomenclature Committee. Amyloid.

[2] Chatani E., Yuzu K., Ohhashi Y., Goto Y. (2021). Current Understanding of the Structure, Stability and Dynamic Properties of Amyloid Fibrils. Int. J. Mol. Sci..

[3] Maskevich A.A., Stsiapura V.I., Kuzmitsky V.A., Kuznetsova I.M., Povarova O.I., Uversky V.N., Turoverov K.K. (2007). Spectral Properties of Thioflavin T in Solvents with Different Dielectric Properties and in a Fibril-Incorporated Form. J. Proteome Res..

[4] Frieg B., Gremer L., Heise H., Willbold D., Gohlke H. (2020). Binding Modes of Thioflavin T and Congo Red to the Fibril Structure of Amyloid-β(1–42). Chem. Commun..

[5] Bagriantsev S.N., Kushnirov V.V., Liebman S.W. (2006). Analysis of Amyloid Aggregates Using Agarose Gel Electrophoresis. Methods Enzymol..

[6] Kushnirov V.V., Alexandrov I.M., Mitkevich O.V., Shkundina I.S., Ter-Avanesyan M.D. (2006). Purification and Analysis of Prion and Amyloid Aggregates. Methods.

[7] Howie A.J. (2019). Origins of a Pervasive, Erroneous Idea: The “Green Birefringence” of Congo Red-stained Amyloid. Int. J. Exp. Pathol..

[8] Yakupova E.I., Bobyleva L.G., Vikhlyantsev I.M., Bobylev A.G. (2019). Congo Red and Amyloids: History and Relationship. Biosci. Rep..

[9] Morris K.L., Serpell L.C. (2012). X-ray Fibre Diffraction Studies of Amyloid Fibrils. Methods Mol. Biol..

[10] Vaquer-Alicea J., Diamond M.I. (2019). Propagation of Protein Aggregation in Neurodegenerative Diseases. Annu. Rev. Biochem..

[11] Santos T.G., Lopes M.H., Martins V.R. (2015). Targeting Prion Protein Interactions in Cancer. Prion.

[12] Zhao D., Tahaney W.M., Mazumdar A., Savage M.I., Brown P.H. (2017). Molecularly Targeted Therapies for P53-Mutant Cancers. Cell. Mol. Life Sci..

[13] Yue X., Zhao Y., Xu Y., Zheng M., Feng Z., Hu W. (2017). Mutant P53 in Cancer: Accumulation, Gain-of-Function, and Therapy. J. Mol. Biol..

[14] Ghosh S., Salot S., Sengupta S., Navalkar A., Ghosh D., Jacob R., Das S., Kumar R., Jha N.N., Sahay S. (2017). P53 Amyloid Formation Leading to Its Loss of Function: Implications in Cancer Pathogenesis. Cell Death Differ..

[15] Torreira E., Moreno-Del Álamo M., Fuentes-Perez M.E., Fernández C., Martín-Benito J., Moreno-Herrero F., Giraldo R., Llorca O. (2015). Amyloidogenesis of Bacterial Prionoid RepA-WH1 Recapitulates Dimer to Monomer Transitions of RepA in DNA Replication Initiation. Structure.

[16] Jiang L.L., Che M.X., Zhao J., Zhou C.J., Xie M.Y., Li H.Y., He J.H., Hu H.Y. (2013). Structural Transformation of the Amyloidogenic Core Region of TDP-43 Protein Initiates Its Aggregation and Cytoplasmic Inclusion. J. Biol. Chem..

[17] Smaldone G., Caruso D., Sandomenico A., Iaccarino E., Focà A., Ruggiero A., Ruvo M., Vitagliano L. (2021). Members of the GADD45 Protein Family Show Distinct Propensities to Form Toxic Amyloid-like Aggregates in Physiological Conditions. Int. J. Mol. Sci..

[18] Carver A., Zhang X. (2021). Rad51 Filament Dynamics and Its Antagonistic Modulators. Semin. Cell Dev. Biol..

[19] Bonilla B., Hengel S.R., Grundy M.K., Bernstein K.A. (2020). RAD51 Gene Family Structure and Function. Annu. Rev. Genet..

[20] Liu L., Malkova A. (2022). Break-Induced Replication: Unraveling Each Step. Trends Genet..

[21] Ogawa T., Yu X., Shinohara A., Egelman E.H. (1993). Similarity of the Yeast RAD51 Filament to the Bacterial RecA Filament. Science.

[22] Yu X., Jacobs S.A., West S.C., Ogawa T., Egelman E.H. (2001). Domain Structure and Dynamics in the Helical Filaments Formed by RecA and Rad51 on DNA. Proc. Natl. Acad. Sci. USA.

[23] Haaf T., Golub E.I., Reddy G., Radding C.M., Ward D.C. (1995). Nuclear Foci of Mammalian Rad51 Recombination Protein in Somatic Cells after DNA Damage and Its Localization in Synaptonemal Complexes. Proc. Natl. Acad. Sci. USA.

[24] Scully R., Chen J., Plug A., Xiao Y., Weaver D., Feunteun J., Ashley T., Livingston D.M. (1997). Association of BRCA1 with Rad51 in Mitotic and Meiotic Cells. Cell.

[25] Pellegrini L., Yu D.S., Lo T., Anand S., Lee M., Blundell T.L., Venkitaraman A.R. (2002). Insights into DNA Recombination from the Structure of a RAD51-BRCA2 Complex. Nature.

[26] Morati F., Modesti M. (2021). Insights into the Control of RAD51 Nucleoprotein Filament Dynamics from Single-Molecule Studies. Curr. Opin. Genet. Dev..

[27] Kryndushkin D.S., Alexandrov I.M., Ter-Avanesyan M.D., Kushnirov V.V. (2003). Yeast [PSI+] Prion Aggregates Are Formed by Small Sup35 Polymers Fragmented by Hsp104. J. Biol. Chem..

[28] Xue C., Lin T.Y., Chang D., Guo Z. (2017). Thioflavin T as an Amyloid Dye: Fibril Quantification, Optimal Concentration and Effect on Aggregation. R. Soc. Open Sci..

[29] Barton J., Sebastian Arias D., Niyangoda C., Borjas G., Le N., Mohamed S., Muschol M. (2019). Kinetic Transition in Amyloid Assembly as a Screening Assay for Oligomer-Selective Dyes. Biomolecules.

[30] Sulatskaya A.I., Rodina N.P., Sulatsky M.I., Povarova O.I., Antifeeva I.A., Kuznetsova I.M., Turoverov K.K. (2018). Investigation of α-Synuclein Amyloid Fibrils Using the Fluorescent Probe Thioflavin T. Int. J. Mol. Sci..

[31] Sivanathan V., Hochschild A. (2012). Generating Extracellular Amyloid Aggregates Using E. Coli Cells. Genes Dev..

[32] Sunde M., Blake C. (1997). The Structure of Amyloid Fibrils by Electron Microscopy and X-ray Diffraction. Adv. Protein Chem..

[33] Chiti F., Dobson C.M. (2017). Protein Misfolding, Amyloid Formation, and Human Disease: A Summary of Progress over the Last Decade. Annu. Rev. Biochem..

[34] Fowler D.M., Koulov A.V., Alory-Jost C., Marks M.S., Balch W.E., Kelly J.W. (2006). Functional Amyloid Formation within Mammalian Tissue. PLoS Biol..

[35] Ramshini H., Parrini C., Relini A., Zampagni M., Mannini B., Pesce A., Saboury A.A., Nemat-Gorgani M., Chiti F. (2011). Large Proteins Have a Great Tendency to Aggregate but a Low Propensity to Form Amyloid Fibrils. PLoS ONE.

[36] Watt B., van Niel G., Fowler D.M., Hurbain I., Luk K.C., Stayrook S.E., Lemmon M.A., Raposo G., Shorter J., Kelly J.W. (2009). N-Terminal Domains Elicit Formation of Functional Pmel17 Amyloid Fibrils. J. Biol. Chem..

[37] Stroud J.C., Liu C., Teng P.K., Eisenberg D. (2012). Toxic Fibrillar Oligomers of Amyloid-β Have Cross-β Structure. Proc. Natl. Acad. Sci. USA.

[38] Palmieri L.C., Melo-Ferreira B., Braga C.A., Fontes G.N., Mattos L.J., Lima L.M.T.R. (2013). Stepwise Oligomerization of Murine Amylin and Assembly of Amyloid Fibrils. Biophys. Chem..

[39] Raderschall E., Bazarov A., Cao J., Lurz R., Smith A., Mann W., Ropers H.H., Sedivy J.M., Golub E.I., Fritz E. (2002). Formation of Higher-Order Nuclear Rad51 Structures Is Functionally Linked to P21 Expression and Protection from DNA Damage-Induced Apoptosis. J. Cell Sci..

[40] Richardson C., Stark J.M., Ommundsen M., Jasin M. (2004). Rad51 Overexpression Promotes Alternative Double-Strand Break Repair Pathways and Genome Instability. Oncogene.

[41] Walsh I., Seno F., Tosatto S.C.E., Trovato A. (2014). PASTA 2.0: An Improved Server for Protein Aggregation Prediction. Nucleic Acids Res..

[42] Garbuzynskiy S.O., Lobanov M.Y., Galzitskaya O.V. (2010). FoldAmyloid: A Method of Prediction of Amyloidogenic Regions from Protein Sequence. Bioinformatics.

[43] Conchillo-Solé O., de Groot N.S., Avilés F.X., Vendrell J., Daura X., Ventura S. (2007). AGGRESCAN: A Server for the Prediction and Evaluation of “Hot Spots” of Aggregation in Polypeptides. BMC Bioinform..

[44] Maurer-Stroh S., Debulpaep M., Kuemmerer N., De La Paz M.L., Martins I.C., Reumers J., Morris K.L., Copland A., Serpell L., Serrano L. (2010). Exploring the Sequence Determinants of Amyloid Structure Using Position-Specific Scoring Matrices. Nat. Methods.

[45] Ahmed A.B., Znassi N., Château M.T., Kajava A.V. (2015). A Structure-Based Approach to Predict Predisposition to Amyloidosis. Alzheimer’s Dement..

[46] Jeyasekharan A.D., Liu Y., Hattori H., Pisupati V., Jonsdottir A.B., Rajendra E., Lee M., Sundaramoorthy E., Schlachter S., Kaminski C.F. (2013). A Cancer-Associated BRCA2 Mutation Reveals Masked Nuclear Export Signals Controlling Localization. Nat. Struct. Mol. Biol..

[47] Rajendra E., Venkitaraman A.R. (2010). Two Modules in the BRC Repeats of BRCA2 Mediate Structural and Functional Interactions with the RAD51 Recombinase. Nucleic Acids Res..

[48] Buisson R., Dion-Côté A.M., Coulombe Y., Launay H., Cai H., Stasiak A.Z., Stasiak A., Xia B., Masson J.Y. (2010). Cooperation of Breast Cancer Proteins PALB2 and Piccolo BRCA2 in Stimulating Homologous Recombination. Nat. Struct. Mol. Biol..

[49] Buchhop S., Gibson M.K., Wang X.W., Wagner P., Stürzbecher H.W., Harris C.C. (1997). Interaction of P53 with the Human Rad51 Protein. Nucleic Acids Res..

[50] Jumper J., Evans R., Pritzel A., Green T., Figurnov M., Ronneberger O., Tunyasuvunakool K., Bates R., Žídek A., Potapenko A. (2021). Highly Accurate Protein Structure Prediction with AlphaFold. Nature.

[51] Furlong R.A., Narain Y., Rankin J., Wyttenbach A., Rubinsztein D.C. (2000). Alpha-Synuclein Overexpression Promotes Aggregation of Mutant Huntingtin. Biochem. J..

[52] Yang H., Hu H.Y. (2016). Sequestration of Cellular Interacting Partners by Protein Aggregates: Implication in a Loss-of-Function Pathology. FEBS J..

[53] Demeyer A., Benhelli-Mokrani H., Chénais B., Weigel P., Fleury F. (2021). Inhibiting Homologous Recombination by Targeting RAD51 Protein. Biochim. Biophys. Acta-Rev. Cancer.

[54] Orhan E., Velazquez C., Tabet I., Sardet C., Theillet C. (2021). Regulation of Rad51 at the Transcriptional and Functional Levels: What Prospects for Cancer Therapy?. Cancers.

[55] Sullivan M.R., Bernstein K.A. (2018). RAD-Ical New Insights into RAD51 Regulation. Genes.

[56] Antony H., Wiegmans A.P., Wei M.Q., Chernoff Y.O., Khanna K.K., Munn A.L. (2012). Potential Roles for Prions and Protein-Only Inheritance in Cancer. Cancer Metastasis Rev..

[57] Park S.K., Park S., Pentek C., Liebman S.W. (2021). Tumor Suppressor Protein P53 Expressed in Yeast Can Remain Diffuse, Form a Prion, or Form Unstable Liquid-like Droplets. iScience.

[58] Lin H.Y., Tan G.Q., Liu Y., Lin S.Q. (2019). The Prognostic Value of Serum Amyloid A in Solid Tumors: A Meta-Analysis. Cancer Cell Int..

[59] Findeisen P., Zapatka M., Peccerella T., Matzk H., Neumaier M., Schadendorf D., Ugurel S. (2009). Serum Amyloid A as a Prognostic Marker in Melanoma Identified by Proteomic Profiling. J. Clin. Oncol..

[60] Biaoxue R., Hua L., Wenlong G., Shuanying Y. (2016). Increased Serum Amyloid A as Potential Diagnostic Marker for Lung Cancer: A Meta-Analysis Based on Nine Studies. BMC Cancer.

[61] Yang M., Liu F., Higuchi K., Sawashita J., Fu X., Zhang L., Zhang L., Fu L., Tong Z., Higuchi K. (2016). Serum Amyloid A Expression in the Breast Cancer Tissue Is Associated with Poor Prognosis. Oncotarget.

[62] Fourie C., Shridas P., Davis T., de Villiers W.J.S., Engelbrecht A.M. (2021). Serum Amyloid A and Inflammasome Activation: A Link to Breast Cancer Progression?. Cytokine Growth Factor Rev..

[63] Lee J., Beatty G.L. (2021). Serum Amyloid A Proteins and Their Impact on Metastasis and Immune Biology in Cancer. Cancers.

[64] Wang C., Iashchishyn I.A., Pansieri J., Nyström S., Klementieva O., Kara J., Horvath I., Moskalenko R., Rofougaran R., Gouras G. (2018). S100A9-Driven Amyloid-Neuroinflammatory Cascade in Traumatic Brain Injury as a Precursor State for Alzheimer’s Disease. Sci. Rep..

[65] Lv Z., Li W., Wei X. (2020). S100A9 Promotes Prostate Cancer Cell Invasion by Activating TLR4/NF-ΚB/Integrin Β1/FAK Signaling. Onco. Targets. Ther..

[66] Markowitz J., Carson W.E. (2013). Review of S100A9 Biology and Its Role in Cancer. Biochim. Biophys. Acta.

[67] Brat D.J., Gearing M., Goldthwaite P.T., Wainer B.H., Burger P.C. (2001). Tau-Associated Neuropathology in Ganglion Cell Tumours Increases with Patient Age but Appears Unrelated to ApoE Genotype. Neuropathol. Appl. Neurobiol..

[68] Stridsberg M., Eriksson B., Lundqvist G., Skogseid B., Wilander E., Öberg K. (1995). Islet Amyloid Polypeptide (IAPP) in Patients with Neuroendocrine Tumours. Regul. Pept..

[69] Bononi P.L., Martinez A.J., Nelson R.B., Amico J.A. (1993). Amyloid Deposits in a Prolactin-Producing Pituitary Adenoma. J. Endocrinol. Investig..

[70] Murphy C.L., Kestler D.P., Foster J.S., Wang S., Macy S.D., Kennel S.J., Carlson E.R., Hudson J., Weiss D.T., Solomon A. (2008). Odontogenic Ameloblast-Associated Protein Nature of the Amyloid Found in Calcifying Epithelial Odontogenic Tumors and Unerupted Tooth Follicles. Amyloid.

[71] Kestler D.P., Foster J.S., Macy S.D., Murphy C.L., Weiss D.T., Solomon A. (2008). Expression of Odontogenic Ameloblast-Associated Protein (ODAM) in Dental and Other Epithelial Neoplasms. Mol. Med..

[72] Chandramowlishwaran P., Sun M., Casey K.L., Romanyuk A.V., Grizel A.V., Sopova J.V., Rubel A.A., Nussbaum-Krammer C., Vorberg I.M., Chernoff Y.O. (2018). Mammalian Amyloidogenic Proteins Promote Prion Nucleation in Yeast. J. Biol. Chem..

[73] Campeau E., Ruhl V.E., Rodier F., Smith C.L., Rahmberg B.L., Fuss J.O., Campisi J., Yaswen P., Cooper P.K., Kaufman P.D. (2009). A Versatile Viral System for Expression and Depletion of Proteins in Mammalian Cells. PLoS ONE.

[74] Kachkin D.V., Khorolskaya J.I., Ivanova J.S., Rubel A.A. (2020). An Efficient Method for Isolation of Plasmid DNA for Transfection of Mammalian Cell Cultures. Methods Protoc..

[75] Sopova J.V., Koshel E.I., Belashova T.A., Zadorsky S.P., Sergeeva A.V., Siniukova V.A., Shenfeld A.A., Velizhanina M.E., Volkov K.V., Nizhnikov A.A. (2019). RNA-Binding Protein FXR1 Is Presented in Rat Brain in Amyloid Form. Sci. Rep..

[76] Sivanathan V., Hochschild A. (2013). A Bacterial Export System for Generating Extracellular Amyloid Aggregates. Nat. Protoc..

