# Supplementary material for: Human RAD51 Protein Forms Amyloid-like Aggregates In Vitro
Source: Int J Mol Sci. 2022 Oct 1;23(19):11657. doi: 10.3390/ijms231911657 (PMC9570251; doi:10.3390/ijms231911657)
Supplement: Supplementary file 1 [file ijms-23-11657-s001.zip › ijms-1907118-supplementary.pdf]

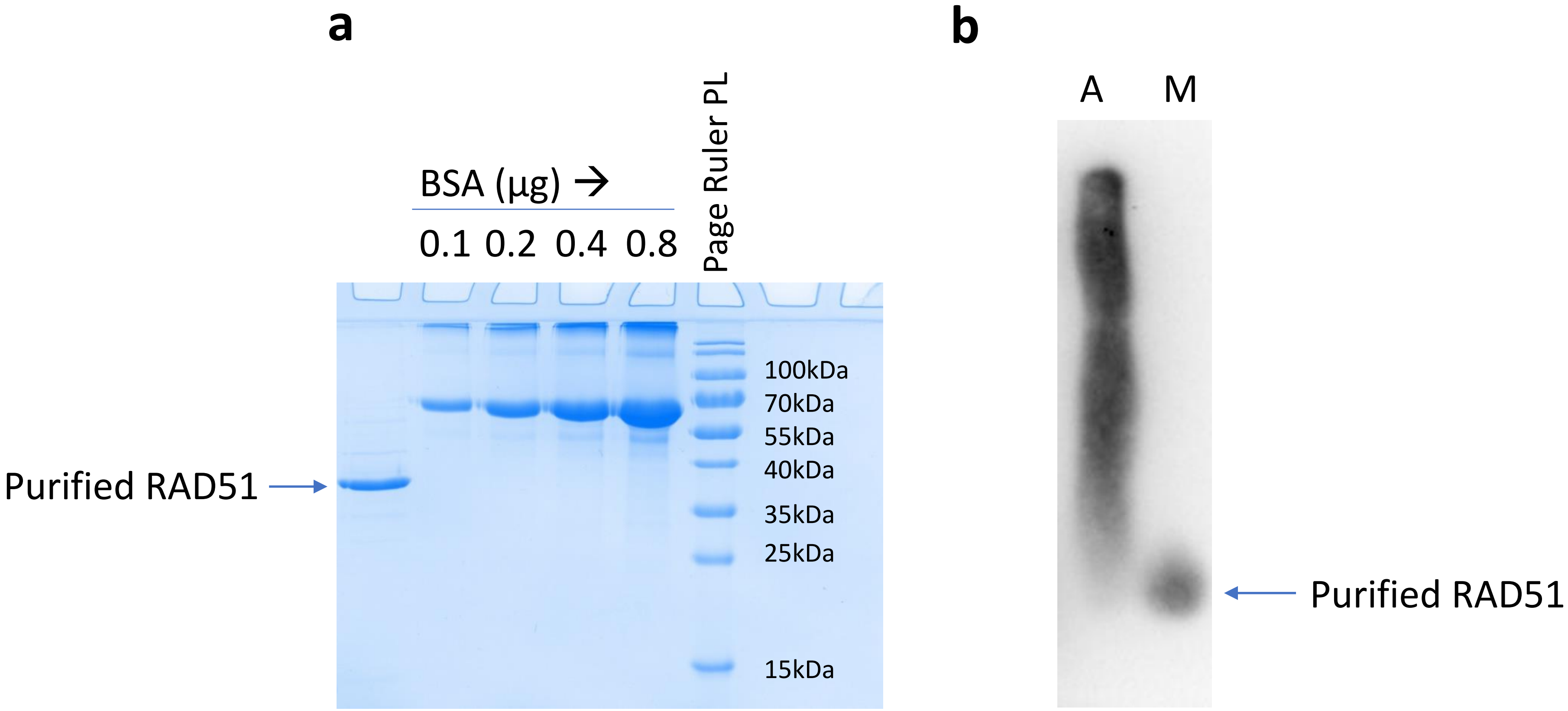

Figure S1. SDS-PAGE and SDD-AGE of purified human RAD51 **(a)** Coomassie-stained PAAG gel (SDS-PAGE) of human RAD51 protein purified from *E.coli* after cleavage of the SUMO-tag, 0.1 µg loaded. PageRuler (Thermo) protein ladder is shown on the left. Different concentrations of BSA were used as loading control. **(b)** SDD-AGE (incubation with 1% SDS) and Western-blot of monomers and aggregates of RAD51. Detection with polyclonal antibodies to RAD51 (ab88572) (Abcam). M – RAD51 monomers (no incubation with agitation), A – RAD51 aggregates.

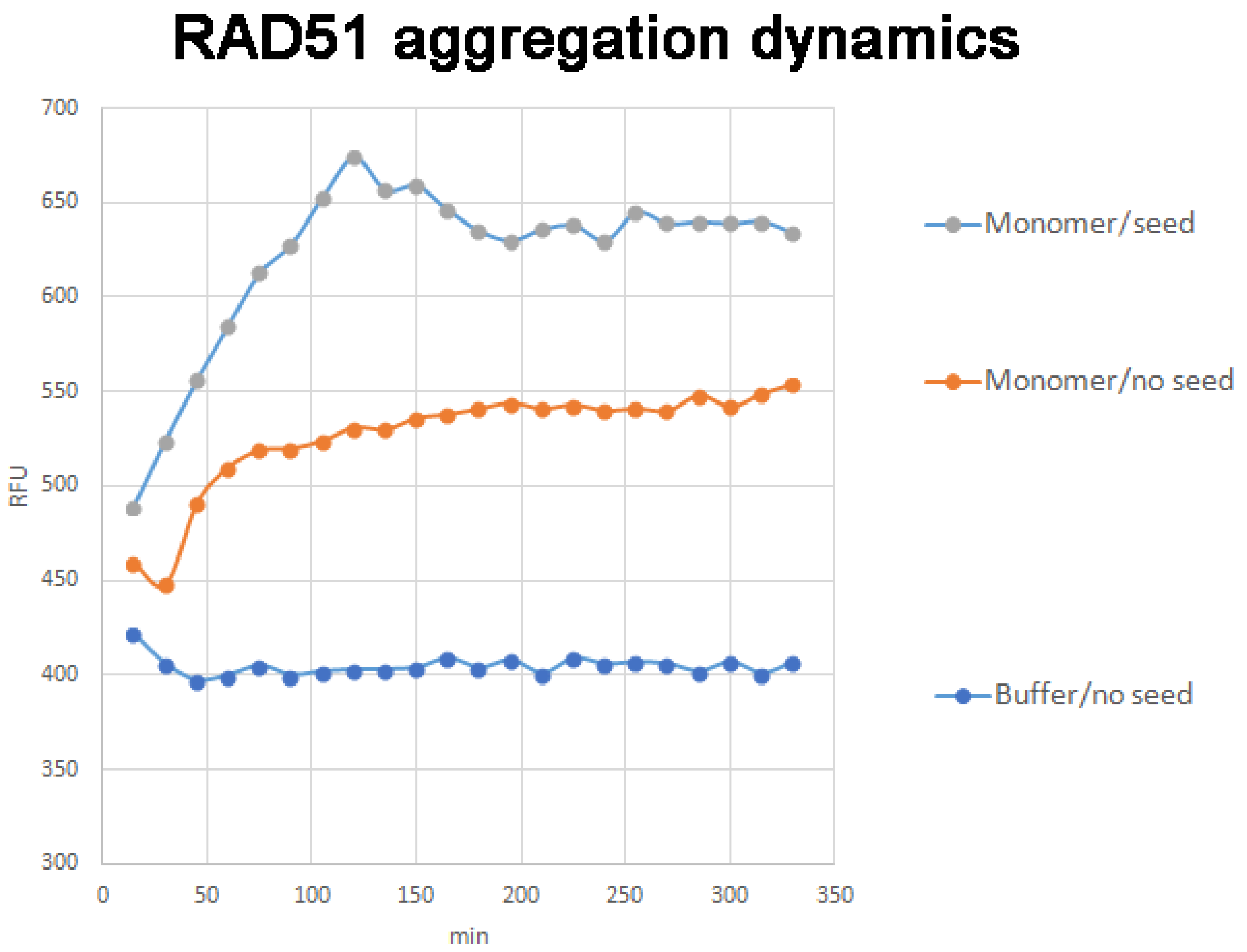

Figure S2. Preformed RAD51 aggregates stimulate the conversion of RAD51 monomers into aggregated state.
RFU – relative fluorescence unit; min – incubation time (minutes); seed – preformed RAD51 aggregates. The experiment is performed as described in Material and Methods.

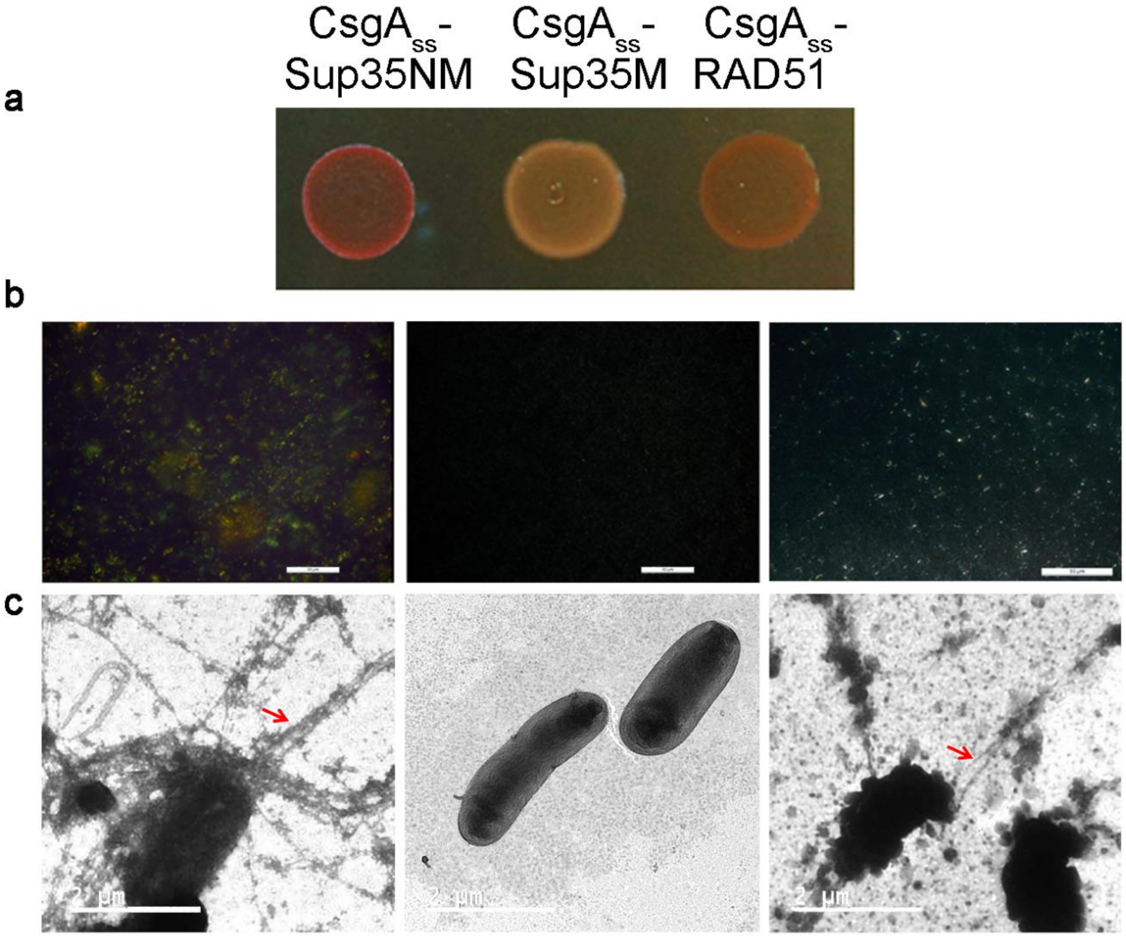

Figure S3. RAD51 shows amyloid properties in the bacteria-based C-DAG system.
(a) *E.coli* cells producing CsgA$_{ss}$-Sup35NM form red colonies, cells producing CsgA$_{ss}$-RAD51 form orange colonies, whereas cells producing CsgA$_{ss}$-Sup35M (negative control) form pale colonies on agar plates containing Congo Red. **(b)** Extracellular material containing CsgA$_{ss}$-Sup35NM and CsgA$_{ss}$-RAD51 binds Congo Red and displays yellow-green birefringence when viewed between crossed polarizers. The negative control (in the center) does not display birefringence. Scale bar, 50 μM **(c)** Secreted CsgA$_{ss}$-Sup35NM and CsgA$_{ss}$-RAD51 proteins form fibrils visualized by transmission electron microscopy. The negative control is in the center. Scale bar, 2μM. Arrows point at amyloid fibrils.

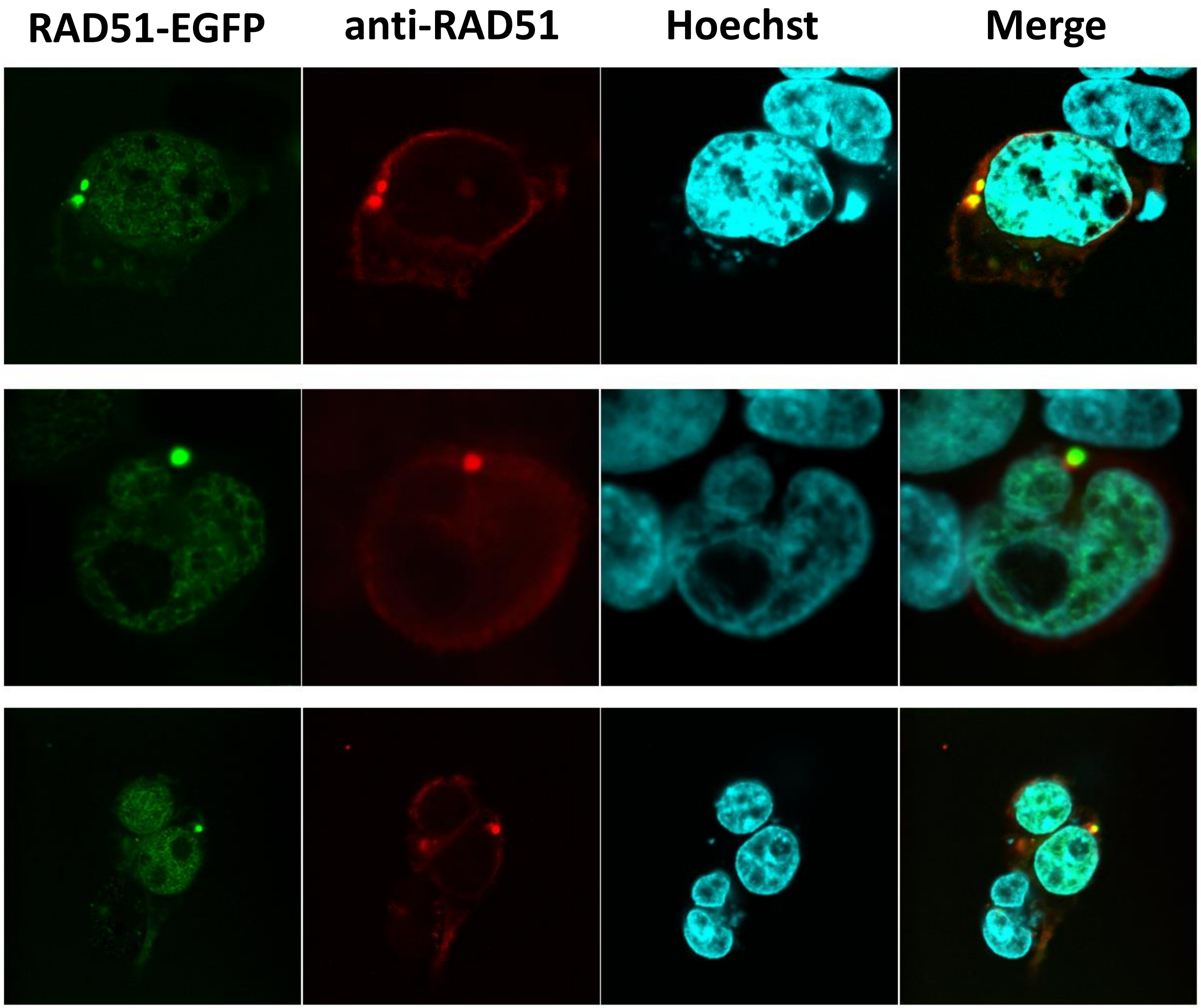

Figure S4. RAD51 aggregation in human cells. Confocal microscopy of HEK293T cells with overexpression of the *RAD51-EGFP*. **RAD51-EGFP** – GFP channel, **anti-RAD51** – detection with primary antibodies for RAD51 (ab88572) and secondary Alexa Fluor 647 (ab150115), **Hoechst** – nuclear staining by Hoechst 33342.
